# Emerging Advancements in Canine and Feline Metabolism and Nutrition

**DOI:** 10.1155/2016/9023781

**Published:** 2016-12-27

**Authors:** Anna K. Shoveller, Maria R. C. De Godoy, Jennifer Larsen, Elizabeth Flickinger

**Affiliations:** ^1^University of Guelph, Guelph, ON, Canada; ^2^University of Illinois, Champaign, IL, USA; ^3^University of California, Davis, CA, USA; ^4^Kent Pet Group, Muscatine, IA, USA

The pet industry continues to grow in both developed and developing countries. According to the 2015-2016 APPA National Pet Owners Survey, 65% of US households own a pet, which encompasses 85.8 million pet cats and 77.8 million pet dogs [1]. In Europe alone, there are another estimated 81 million dogs and 63 million cats, and this market is expected to have a compound annual growth rate of 4.4%, as compared to a 3.5% projected growth rate in the USA [2]. Much of this growth has been stimulated by the increasing appreciation of the value of pets to human health and well-being through both physical and emotional effects. Studies indicate associations between pet ownership and(or) animal-assisted therapy and numerous aspects of positive health outcomes, ranging from improved cardiovascular health to enhanced mental well-being [3, 4]. Humans, reciprocally, are engaged in gaining a deeper understanding of nutrient requirements and the effects of diet and care practices on the health, metabolism, and behavior of cats and dogs of all ages, breeds, and lifestyles. This special issue adds to the primary literature concerning canine and feline metabolism, nutrition, and behavior.

Vitamin A has long been known to be essential for life for all vertebrates, and the review by A. S. Green and A. J. Fascetti included in this issue provides a concise summary of the research available to date across multiple species. As the authors point out, we still have much to learn about the metabolism of the most important vitamin A precursor, *β*-carotene. Dogs can meet their entire vitamin A requirement through *β*-carotene [5]. And although cats require preformed vitamin A, they can efficiently absorb *β*-carotene, as evidenced by elevated plasma concentrations after supplementation [6]. Furthermore, *β*-carotene may play additional roles in health, including immune response, gap junction communication, and other cellular functions [7], but more research is needed to further elucidate these effects.

In addition to companions, dogs long have been employed for work and admired for their athleticism. Ultramarathon sled dog races are an excellent model for studying metabolism under extreme energy demands. The original research conducted by M. W. Brunke et al. included in this issue reports how concentrations of insulin-like growth factor-1 and its binding proteins fluctuate in response to exercise and negative energy balance. These data give important evidence on how to better fuel these athletes and highlight the need for further research into the dietary needs of canine athletes in order to optimize health and well-being as well as maximize performance.

Most dogs and cats, however, have a sedentary lifestyle, which is correlated with the rise in pet obesity [8]. The study by S. A. Sapowicz et al. underscores how pervasive obesity is regardless of socioeconomic status and how the role of nutrition in promoting healthy weight is poorly understood by pet owners. Dietary approaches to combat obesity commonly include feeding low fat, high fiber, and(or) low carbohydrate diets, as well as using nutraceutical additives [9]. One such nutraceutical, L-carnitine, was studied by M. A. Gooding et al. in the diet of cats. The results included in this issue suggest that higher doses of L-carnitine may attenuate body fat accumulation when cats are free fed.

In addition to nutrition and feeding practices, understanding pet owners' management practices is vital for promoting pet welfare. In particular, research in cat behavior is lacking. This issue adds two original works conducted by J. L. Stella and C. C. Croney. Behavioral problems are a leading reason for cat relinquishment in the USA [10]. An underlying factor may well be that cats' needs are inadequately met in the home environment. These papers outline the key considerations for cat environmental management and reveal that a sizeable proportion of homes do not implement best practices for cat care.

Clearly, more research is needed in canine and feline nutrition, metabolism, and behavior. While much work has been done to elucidate nutrient requirements of adult dogs and cats at maintenance, a paucity of information exists with regard to the role of nutrient precursors or requirements in exercising animals. In addition, the pet obesity epidemic has continued to grow despite research in this area, so novel approaches are needed. Furthermore, nutrition can greatly impact health, but, for a truly holistic approach, it is necessary to also provide excellent animal care in an appropriate environment. A deeper understanding of companion animal nutrition, metabolism, and behavior will allow improvements in diet formulation and management practices and will ultimately lead to a better quality of life and perhaps even longer life for dogs and cats. Ultimately, improvements in the life of pets will have beneficial effects on the families that these pets are part of.

*Anna K. Shoveller* *Maria R. C. De Godoy* *Jennifer Larsen* *Elizabeth Flickinger*
